# *Zingiber zerumbet* (L.) Smith: A Review of Its Ethnomedicinal, Chemical, and Pharmacological Uses

**DOI:** 10.1155/2011/543216

**Published:** 2011-03-22

**Authors:** N. J. Yob, S. Mohd. Jofrry, M. M. R. Meor. Mohd. Affandi, L. K. Teh, M. Z. Salleh, Z. A. Zakaria

**Affiliations:** ^1^Department of Pharmaceutics, Faculty of Pharmacy, MARA University of Technology, Puncak Alam Campus, Bandar Puncak Alam, 42300 Selangor, Malaysia; ^2^Pharmacogenomics Center, Faculty of Pharmacy, MARA University of Technology, Shah Alam, 40450 Selangor, Malaysia; ^3^Department of Biomedical Sciences, Faculty of Medicine and Health Science, Putra Malaysia University, UPM Serdang, 43400 Selangor, Malaysia

## Abstract

*Zingiber zerumbet* Sm., locally known to the Malay as “Lempoyang,” is a perennial herb found in many tropical countries, including Malaysia. The rhizomes of *Z. zerumbet*, particularly, have been regularly used as food flavouring and appetizer in various Malays' cuisines while the rhizomes extracts have been used in Malay traditional medicine to treat various types of ailments (e.g., inflammatory- and pain-mediated diseases, worm infestation and diarrhea). Research carried out using different *in vitro* and *in vivo* assays of biological evaluation support most of these claims. The active pharmacological component of *Z. zerumbet* rhizomes most widely studied is zerumbone. This paper presents the botany, traditional uses, chemistry, and pharmacology of this medicinal plant.

## 1. Introduction

Considered as a moderately large genus of herbs belonging to the family Zingiberaceae, genus *Zingiber *is represented by approximately 141 species that are distributed mainly in Asia. This genus of plant is confined to the tropics of Asia, Malaysia, and the Pacific Islands [1, 2]. The name *Zingiber*, actually derived from a Sanskrit word that refers to bull's horn [3]. One particular plant of this genus that has gained much interest from scientists all over the world because of its high medicinal values is *Zingiber zerumbet *(L.) Smith*. Z. zerumbet*, commonly known as the pinecone or shampoo ginger, is a perennial, tuberous root herb plant that can be found growing naturally in damp, shaded parts of the lowland or hill slopes, as scattered plants or thickets. It is known by various names, for example, “*Lempoyang*” (Malaysia and Indonesia), “*Ghatian*” and “*Yaiimu*” (India), “*Jangli adha*” (Bangladesh), “*Awapuhi*” (Hawaii), “*Zurunbah*” (Arab), “*Hong qiu jiang*” (China), and “*Haeo dam*” or “*Hiao dam*” (Northern Thailand) [4–8]. This herbal plant is believed to be native to India and the Malaysian Peninsula, and since it has been cultivated for so long in so many places throughout Southeast Asia, the Pacific, and Oceania, it became uncertain as to where the plant had originated. *Z. zerumbet* has also been claimed to be introduced throughout the Pacific by the ancient Polynesian settlers [9]. 

**Figure 1:**
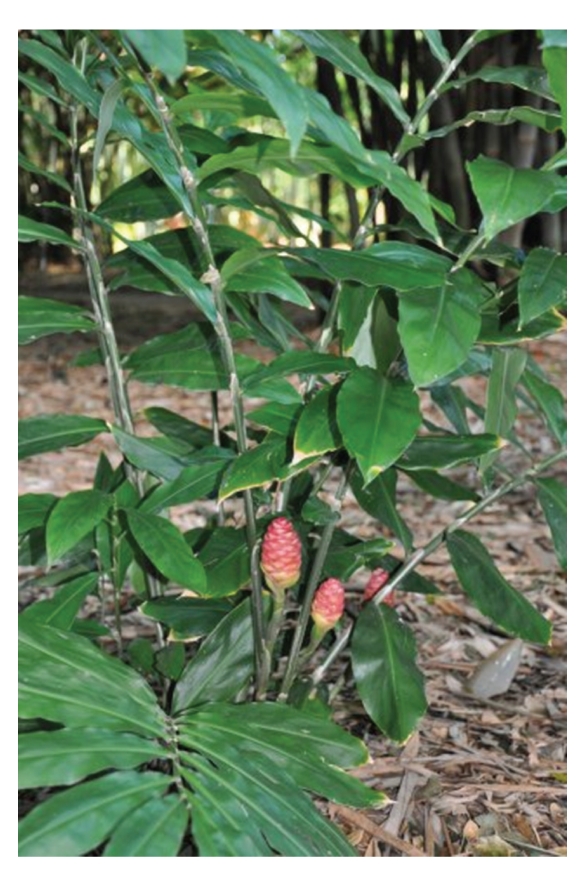
The leaves and inflorescences of *Z. zerumbet*.

**Figure 2:**
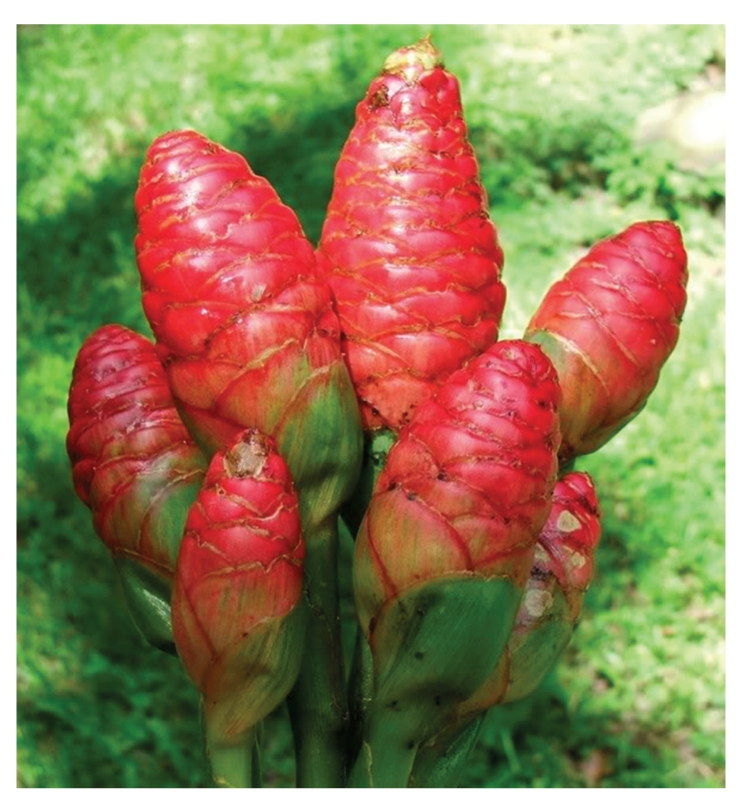
The inflorescences of *Z. zerumbet*.

**Figure 3:**
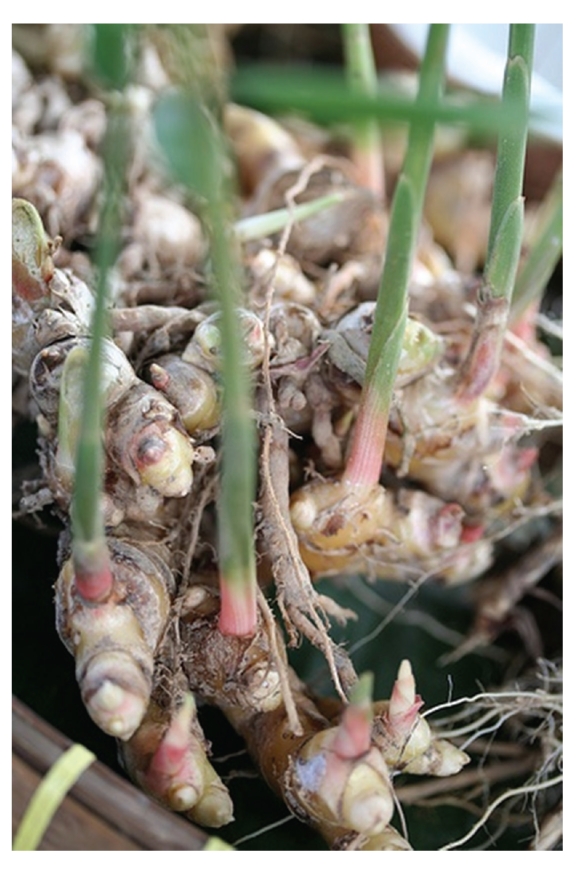
The rhizomes of *Z. zerumbet*.

*Z. zerumbet* can be characterized by the presence of a pulvinus between the base of the petiole and ligule [10] and is a variegated wild edible ginger with stems of approximately 1-2 m tall that are erect, oblique, round, annual and invested by the smooth sheaths of the leaves. The leaves and inflorescences of the pinecone ginger crop up from a thick knobbly rhizome or the underground stem that grows just under the surface of the soil (Figure 1). The leaves, which are sometimes purplish beneath young shoots, are thin approximately 25–35 cm long with their midribs strongly raised on the lower surface. The petiole is approximately 6 mm long while the ligule, which is very thin, entire, and broad, is approximately 1.5–2.5 cm long. The leaflets are arranged alternately along an arching pseudostem that grows 1-2 m in length. The inflorescence, which is approximatly 6–12 cm long and green when young and becomes red when old, is borne on a separate pseudostem from the leaves and has closely overlapping bracts or bracts that form an open pouch in which flowers occur, one in each bract (Figure 2). It is a spike, ovoid to ellipsoid in shape; bracts subtend the position of each of the flowers giving the inflorescence its pinecone shape. *Z. zerumbet* is also known as shampoo ginger because the mucilaginous substance present in the inflorescence is used by the Hawaiians as shampoo and natural hair conditioner [10, 11]. The bracts are approximately 3–3.5 cm long and 2.5 cm wide while the bracteole is approximately 2.5 cm long, wide and thin but persistent until fruiting. The pale yellow or white flowers, which are usually longer than bracts, fragile, ephemeral and only lasting a few hours, produce in August and September [11] and emerge from the lowest bracts first, and when exhausted, the flower dries and falls away. The lip of the flower is three lobed. After flowering, the bracts change colour, which continues upward until the entire inflorescence is bright crimson. The corolla tube is as long as the bract while the style is long and filiform. The stigma is slightly projecting and its margin is ciliate. The unique characteristic of *Z. zerumbet *and other plant within this genus is that the stamen, which dehisces longitudinally, is attached with a long curved beak or horn-like appendage. One stamen of the inner whorl is fertile, and the two staminoids have petal-like shape. The ovary is inferior and trilocular with axile placentation. Fruit is white, glabrous, thin walled and approximately 1.5 cm long. The seeds, which are covered with lacerate aril, are ellipsoid and black [10, 12]. The most important part of *Z. zerumbet *is its rhizomes, which have been associated with all the claimed medicinal uses. The rhizome of *Z. zerumbet*, which is perennial, thick, scaly, aromatic, and pale yellow internally (Figure 3), is used exclusively by this obligates asexual reproducer plant for propagation. 

The rhizome of *Z. zerumbet* (RZZ), in particular, has been used traditionally as herbal medicine in Asian, Indian, Chinese, and Arabic folklores since ancient times. Despite its regular uses as food flavoring and appetizer in Malays and Indian cuisines, the rhizomes of *Z. zerumbet*, in particular, have also been used in folkloric medicine as a cure for various ailments [13–15]. Many reviews have appeared in the literature regarding *Z. zerumbet* particularly on its medicinal uses but none have described the complete chemical and pharmacological properties of this important ethnomedicinal plant. Therefore, we aimed to compile an up-to-date and comprehensive review of *Z. zerumbet* that covers its ethnomedicinal uses, phytochemical contents, and pharmacological activities.

## 2. Ethnomedicinal/Traditional Uses

Various ginger spices of the Zingiberaceae family are widely used as spices, flavouring agents, and medicines in Southeast Asia because of their unique flavour as well as due to their medicinal properties. Some of the RZZ traditional usages as botanical medicine include the treatment of inflammation, fever, toothache, indigestion, constipation, diarrhea, severe sprains, and to relieve pain, as well as antispasmodic, antirheumatic, and diuretic agents [5–7, 14, 16]. Other than that, the Malays used the fresh rhizomes as a cure for edema, stomach ache, sores, and loss of appetite while the juice of the boiled rhizomes is used to treat worm infestation in children [1, 14, 17, 18]. In Thailand, the fresh rhizomes are also used as antiflatulent agent. Meanwhile, the Chinese macerated the rhizomes in alcohol and use it as a tonic, depurative, or stimulant while the Taiwanese used the plant as an anti-inflammatory adjuvant for stomach ache, sprain, and fever. In India, the rhizome powder is mixed with ripe *Morinda citrifolia *for the treatment of severe pain, the cooked and softened rhizome is used to treat toothache, cough, asthma, worms, leprosy, and other skin diseases, and the ground and strained rhizome is mixed with water and drank to treat stomach ache [7]. The Hawaiians apply the compressed RZZ to sore spots, bruises, and cuts and also used it to treat headaches, toothache, ringworm/other skin disease, achy joints/sprains, stomach-ache. In addition, they also used ashes from burnt *Z. zerumbet *leaves, which are combined with a mixture of ashes of *Schizostachyum glaucifolium*, nut sap of *Aleurites moluccana*, and tuber sap of* Z. zerumbet*, as a remedy for cuts and bruised skin while the RZZ was mashed with salt and rubbed on the head to treat headaches [19, 20]. Furthermore, the plant's pine cones are used as an ornamental in gardening, and the milky juice obtained from the pine cones is famously used as a shampoo in Hawaii [21].

## 3. Phytochemical Contents

Of all parts of the plant, the RZZ has been the subject of extensive chemical investigations because of its high medicinal values. Various reports have been published regarding the phytochemical content of RZZ. Attempts to isolate and identify bioactive compounds from the RZZ had started since 1944 with the identification of humulene [22], monoterpenes [23, 24], and zerumbone (2,6,10-cycloundecatrien-1-one, 2,6,9,9-tetramethyl-,(E,E,E)-) [24] from the essential oil of RZZ (EOZZ). This is followed by the success of Ramaswami and Bhattacharyya [25] in identifying two new oxygenated derivatives of humulene (humulene monoxide and humulene dioxide) and Nigam and Levi [26] in reporting the presence of 8 fractions when the EOZZ was subjected to the column chromatography with petroleum ether, benzene, ether, and methanol used as eluants. Those fractions were reported to contain *α*-pinene, *β*-pinene, Δ^3^-carene, camphor, *β*-caryophyllene, ar-curcumene, zerumbone, humulene oxide, humulene dioxide, linalool, borneol, *α*-terpineol, unidentified sesquiterpene ketones and sesquiterpene alcohol. Meanwhile Damodaran and Dev [27, 28] reported the isolation of new sesquiterpenoids (e.g., humulene epoxide-I, humulene epoxide-II, humulenol-II, dihydro-*ψ*-photo-zerumbone and *ψ*-photozerumbone) and sesquiterpene alcohols (humulenol-I and humulenol-III) from the sesquiterpene fractions and EOZZ, respectively. Chhabra et al. [29] have successfully isolated zerumbone oxide from RZZ while Matthes et al. [30] managed to isolate one new (3′′,  4′′-0-diacetylafielin) and five known compounds (zerumbone, zerumbone epoxide, diferuloylmethane, feruloyl-p-coumaroyl-methane and di-p-coumaroyl-methane) from pentane and ether extracts of RZZ, respectively. Masuda et al. [31] have successfully isolated three new acetylated (3-*O*-(2-*O*-acetyl-*α*-L-rhamnopyranoside), 3-*O*-(3-*O*-acetyl-*α*-L-rhamnopyranoside),  3-*O*-(4-*O*-acetyl-*α*-L-rham-nopyrano-side)) and one known (3-*O*-*α*-L-rhamnopyranoside) kaempferol glycosides from the acetone extract of the fresh RZZ. Dung et al. [32, 33] reported high proportions of (Z)-nerolidol (22–36%) in extracts of stems, leaves, and flowers, but not rhizomes, and found zerumbone to predominate in leaves. Further phytochemical investigations demonstrated the presence of sesquiterpenoids, flavonoids, aromatic compounds (e.g., hydroxybenzaldehyde), vanillin and kaempferol derivatives in the RZZ [34–36]. The ethyl acetate fractions of the RZZ (EAZZ) have been reported to contain kaempferol-3-*O-*rhamnoside, kaempferol-3-*O-*(2′′- or 3′′-acetyl) rhamnoside, kaempferol-3-*O-*(4′′-acetyl) rhamnoside, kaempferol-3-*O-*(3′′,  4′′-diacetyl) rhamnoside and kaempferol-3-*O-*(2′′,  4′′-diacetyl) rhamnoside [18], which concur with report by Lako et al. [37] on the presence of high content of kaempferol in the RZZ. All reports mentioned above were further confirmed by findings of Srivastava et al. [38] and Yu et al. [39] who reported the presence of approximately 86% sesquiterpenoids with zerumbone being the major component in the EOZZ. Yu et al. [39] also reported the presence of *β*-caryophyllene, caryophyllene oxide and *β*-eudesmol in the EOZZ. Besides having zerumbone as its main constituent, earlier study also reported that the EOZZ has characteristics of being dextrorotatory and congeals at 3°C [40]. In contrast to these findings, the EOZZ of another proposed variety of *Z. zerumbet* was characterized as being levorotatory, congeals at −27°C and contains 4-terpinenol as its main constituent. In a latest study, attempt to identify anti-inflammatory compounds from the rhizome of *Z. zerumbet *led to isolation of one sesquiterpene (zerumbone), one flavone (3-*O*-methyl kaempferol), and two flavonoid glycosides (kaempferol-3-O-(2,4-di-*O*-acetyl-*α*-L-rhamnopyranoside) and kaempferol-3-O-(3,4-di-*O*-acetyl-*α*-L-rhamnopyranoside)) from the methanol extract of *Z. zerumbet* (MEZZ) [36]. According to Dae et al. [34] study on the chloroform soluble fraction of *Z. zerumbet* led to the isolation and identification of two aromatic compounds, p-hydroxybenzaldehyde and vanillin, and six kaempferol derivatives. Chane-Ming et al. [41] reported the EOZZ was rich in zerumbone, *α*-pinene, and camphene. In contrast, the oils from leaves and flowers contained high amount of (E)-nerolidol, *β*-caryophyllene, and linalool, respectively, with the former differed from the others by their high levels of *α*- and *β*-pinene. In addition to these, leaves and flowers also contain zingiberene [5]. Interestingly, in most of the studies that described the presence of zerumbone, the compound has been reported as the predominant compound in RZZ. Recent study revealed the presence of a sesquiterpene, zederone, in ethanol extract of RZZ (EEZZ) [42]. Phytochemical screening to the aqueous extract of RZZ (AEZZ) was reported to contain phenolic, saponins, and terpenoids [43].

## 4. Pharmacological Properties

Various studies have revealed the different pharmacological potentials of *Z. zerumbet* in a range of *in vitro *and *in vivo *test models. The RZZ, in particular, has been demonstrated to possess anti-inflammatory, antinociceptive, antiulcer, antioxidant, anticancer, antimicrobial, antihyperglycemic, antiallergic, antioxidant, and antiplatelet activities at different doses/concentrations. These have been described in greater detail in the following subsections.

### 4.1. Acute Toxicity

The AEZZ at 2000 mg/kg body weight did not cause any behavioral changes to the broiler chickens immediately after its administration indicated by normal movement and drinking behavior [43]. However, after 1 hour the extract-treated birds exhibited signs of inappetence which lasted until 24 hours after the extract administration. Interestingly, no mortality was recorded until 14 days of the extract administration while histopathological observation of the livers and kidneys at macroscopic and microscopic levels indicated no alteration to the treated organs. The clinical blood chemistry examination indicated significant increases in the serum level of ALT, ALP and AST of female broiler after 7 and 14 days of treatment with the 2000 mg/kg body weight AEZZ when compared to the control group. The serum values of ALT, ALP and AST at 0, 7 and 14 days after the extract administration were 2.94 ± 0.12, 11.10 ± 1.06, and 6.84 ± 0.15; 2168 ± 170, 2580 ± 172, and 3117 ± 155; 202 ± 14.83, 279 ± 13.04, and 198 ± 2.36 as compared to the control group (2.68 ± 0.14, 5.34 ± 1.14, and 5.66 ± 0.15; 2224 ± 143, 3339 ± 323, and 2531 ± 196; 202 ± 11.56, 263 ± 13.11, and 215 ± 14.62, resp.). Furthermore, the serum total protein, albumin, globulin, creatinine, and urea level of extract-treated broiler was not significantly different when compared to the control group when measured immediately and 7 days after the extract or vehicle administration. However, 14 days after the test solutions administration, only the serum level of urea increased significantly in the broilers treated with AEZZ extract. The effects of 2000 mg/kg AEZZ on broilers' feed intake, body weight gain and feed conversion ratio were also measured whereby the extract significantly increased the body weight of broilers at 7 and 14 days after its administration. The extract was also found to significantly increase food consumption until the end of the experiment (35 days) when compared to the control group.

### 4.2. Antinociceptive Activity

Somchit et al. [44] have earlier reported on the antinociceptive profiles of AEZZ and EEZZ administered intraperitoneally into rats and assessed using the 0.6% acetic acid-induced writhing test. In this study, only the EEZZ was found to be significantly (*P* < .05) effective in reducing the number of writhing in a dose-dependent manner. The activity was observed at the lowest dose (10 mg/kg) 20 min after the phlogistic agent administration in rats and lasted until the end of experiment (40 min). Morphine, at the doses of 2 and 8 mg/kg, was used as the standard reference drugs. Interestingly, the 25 and 100 mg/kg EEZZ antinociceptive activity was comparable to that of 2 and 8 mg/kg morphine, respectively. 

Recent studies using the MEZZ (25, 50, and 100 mg/kg; given subcutaneously) and EOZZ (30, 100, and 300 mg/kg; given intraperitoneally) demonstrated that the extract and oil exhibited significant (*P* < .05) antinociceptive activity at the peripheral and central levels when assessed using the writhing, hot plate, and formalin tests [6, 14]. Interestingly, the antinociceptive activity of MEZZ and EOZZ was attenuated by 5 mg/kg naloxone indicating that the antinociceptive activity observed was modulated partly via the opioid receptor system. The ability of both MEZZ and EOZZ to inhibit nociception in the hot plate test and in both neurogenic and inflammatory phases of the formalin test suggested that the antinociceptive activity involved peripheral and central mechanism of actions. Comparison of extract's antinociceptive activity was made against 100 mg/kg ASA and 5 mg/kg morphine, which represent the peripherally and centrallyacting antinociceptive agent, respectively. ASA produced approximately 63% antinociception in the writhing test when compared against the MEZZ that gave range between 17–55% antinociception. Morphine, on the other hand, exerted high antinociceptive activity as indicated by increase of latency against discomfort 90 min after its administration while 100 mg/kg MEZZ exerted high antinociceptive activity 120 min after its administration. Furthermore, ASA and morphine produced 8% and 63% antntinociception in the first phase and 67% and 23% antinociception in the second phase of the formalin test. In comparison, MEZZ produced approximately 27–45% and 34–62% antinociception in both phases, respectively.

### 4.3. Anti-Inflammatory Activity

The anti-inflammatory profile of intraperitoneally administered AEZZ and EEZZ, at the doses of 25–100 mg/kg, against prostaglandin-E2-(PGE_2_-) induced paw edema test has been reported earlier by Somchit and Nur Shukriyah [17]. The AEZZ exhibited significant anti-inflammatory activity only at the doses of 50 and 100 mg/kg with the former dose producing an activity between 1–4 h while the latter exhibiting an activity between 0.5–4 h after PGE_2_ administration. On the other hand, the EEZZ failed to affect the PGE_2_-induded paw edema at all doses tested. Mefenamic acid, at the dose of 20 mg/kg, was used as standard drug and produced peak effects of approximately 50% inflammatory inhibition for the first 2 h after its administration before being constantly reduced until it reached 30% inhibition at the end of the experiment (at interval time of 4 h). The AEZZ with percentage of inflammatory inhibition of approximately 50% was found to produce anti-inflammatory activity that was comparable to that of mefenamic acid for the first 2-hour-interval.

The MEZZ (25–100 mg/kg; given subcutaneously) also demonstrated significant (*P* < .05) antiedema and anti-inflammatory activities when assessed using the acute (carrageenan-induced paw edema test) and chronic (cotton-pellet-induced granuloma test) models of inflammation, respectively [6]. All dosages of MEZZ exhibited significant antiedema activity in the paw edema test in a concentration-dependent manner. The onset of antiedema effect of 50 and 100 mg/kg MEZZ was 1.5 h after the phlogistic agent administraton while for 25 mg/kg extract, the onset of antiedema was 2.5 h after the carrageenan administration. Overall, the antiedema activity of MEZZ, which lasted until the end of experiment (6.5 h), was lower than 100 mg/kg ASA. In addition, all doses of MEZZ also significantly reduced the weight of exudates and granuloma tissues in the granuloma test in a dose-dependent manner. The 50 mg/kg MEZZ caused 47% reduction in the weight of exudates and 52% reduction in granuloma tissues. Additional study using the isolated rat's ileum preparation demonstrated that MEZZ also affected the organ contraction induced by bradykinin, PGE_2_ and histamine [6]. Further study using the EOZZ also revealed the oil potential as anti-inflammatory agent based on its ability to attenuate inflammation induced in both tests mentioned earlier when given intraperitoneally [7]. Interestingly, the EOZZ was also reported to attenuate the second phase of the formalin-induced pain test, which is associated with inflammatory-mediated pain [7]. In both reports [6, 7] 100 mg/kg ASA was used as a standard reference drug in carrageenan-induced paw edema and cotton-pellet-induced granuloma tests. The ASA exerted significant anti-inflammatory activity that was comparable to 100 mg/kg MEZZ in the paw edema tests and to 50 and 100 mg/kg MEZZ in the granuloma test. The ASA also exerted greater anti-inflammatory activity against the 30, 100, and 300 mg/kg EOZZ in the first 3 h after its administration in the paw edema test, but this activity reduced significantly for the next 2 h at which the 100 and 300 mg/kg EOZZ activity increased remarkably. In the granuloma test, the 100 mg/kg ASA produced approximately 60% antitransudative and antiproliferative activities. In comparison, the 30–300 mg/kg EOZZ produced both activities in the range of 60–75% and 42–66%, respectively.

### 4.4. Antipyretic Activity

The intraperitoneally administered AEZZ and EEZZ, at the doses of 25, 50, and 100 mg/kg, were reported to exert antipyretic activity when assessed for 8 h using the Brewer's yeast-induced pyrexia test [44]. Both extracts demonstrated dose-dependent antipyretic effect with the EEZZ significantly reducing the rat's rectal temperature only at the doses of 50 and 100 mg/kg while the AEZZ was effective at all doses used. Unfortunately, the antipyretic potential of both extracts was not compared against any standard reference drugs.

### 4.5. Hepatoprotective Activity

Further study on hepatotoxicity potential of RZZ demonstrated that the crude AEZZ, at the doses of 50 and 500 mg/kg, did not cause hepatotoxicity effect in mice, which concurred with the insignificant changes in the serum level of ALT and AST after treatment for 4 weeks [45]. Although the AEZZ did cause elevation in serum creatinine level, there are no pathological renal disorders seen. This is further supported by the insignificant changes in the serum blood-urea-nitrogen (BUN) levels between the parallel control (PC) and treated mice indicating normal renal function. In anti-inflammatory study, the AEZZ significantly inhibited the release of TNF-*α* in murine peritoneal macrophages following LPS stimulation, which occurred in a dose-dependent manner, and TNF-*α* expression by 55% and 38%, respectively. The AEZZ was also found to inhibit inteleukin-4 (IL_4_) production in EL-4 lymphocytes at the dose of 500 *μ*g/mL. Compounds like 6-gingerol, kaempferol glycosides and kaempferol derivatives, and chromenone, but not zerumbone, were suggested to contribute to the observed anti-inflammatory activity of the crude AEZZ. The authors suggested that zerumbone was not one of them as it did not dissolve easily in water-soluble fractions. In antipulmonary inflammatory study, the AEZZ was also found to significantly reduce cysteinyl leukotriene (LTC_4_) (39% reduction) release by the lung tissue of treated mice when compared to the control mice (429 ± 69 versus 261 ± 23 *ρ*g/lung) without affecting the serum level as interleukin-1*α* (IL_1*α*_) suggesting that the extract is not inducing any inflammatory effect upon long-term oral administration to mice and is not causing any harmful effect to the liver or to renal function. LTC_4_ plays a significant role in the pathophysiology of asthma. These findings suggested the potential of AEZZ in improving symptoms of asthma without exacerbating inflammatory reactions. In *in vivo* anti-hypersensitivity study, the AEZZ as well as PC group caused significant increase in mRNA expressions of both IL_4_ and IFN_*γ*_ in the splenocytes of mice induced with allergic inflammation when compared to control group. Moreover, the AEZZ as well as the PC group also increased gene expressions of IL_4_ and IFN_*γ*_ levels that were 3, 50, 11, and 12 times, respectively, when compared to the normal group in the ConA-stimulated splenocytes. In mice not stimulated with ConA, the gene expressions of IL_4_ and IFN_*γ*_ levels increased about 30-, 4-, 12-, and 20-fold in extract-treated mice and PC group compared to the NC group, respectively. Overall, the AEZZ was found to significantly increase gene expression of interferon-*γ* (IFN_*γ*_) while significantly reducing the expression of IL_4_ gene expression in splenocytes stimulated with ConA or without ConA, whereby both mediators are involved in the regulation of immune response during human asthmatic attacks. In addition, the AEZZ also increased the ratios of splenocyte IFN_*γ*_/IL_4_ gene expression by 5- and 12-fold when compared to the ratios of NC group, which suggested that the allergic asthmatic reactions might be eliminated via long-term upregulation of the T-helper1/T-helper2 (Th1/Th2) response during the modulation of the cytokine gene expression in immune cells. These findings revealed the potential of RZZ to exert anti-inflammatory and anti-hypersensitive activities via its ability to inhibit the synthesis of LTC4 and through the immunomodulation of Th1/Th2 cytokine production.

### 4.6. Antiallergic Activity

The EEZZ and AEZZ and volatile oil of RZZ (VOZZ) were subjected to an *in vitro* investigation for their antiallergic activity [46]. The extracts and oil, at the concentrations of 0–100 *μ*g/mL, were investigated for its ability to inhibit the release of *β*-hexosaminidase from RBL-2H3 cell line. The EEZZ and AEZZ inhibited the release of *β*-hexosaminidase from the cells between 10–100 *μ*g/mL with inhibitory percentage of 8.4–53.7% and 10.9–59.1%, respectively. The IC_50_ value obtained for both extracts was 91.0 and 68.2 *μ*g/mL, respectively. In comparison, the standard drug, ketotifen fumarate, at doses ranging between 0–100 *μ*g/mL, exhibited inhibitory percentage ranging between 0–68.2% with IC_50_ value of 20.2 *μ*g/mL (47.5 *μ*M). On the other hand, the VOZZ failed to inhibit *β*-hexosaminidase at all concentrations tested. The EEZZ, AEZZ, and VOZZ were also found to be inactive against the enzyme activity of *β*-hexosaminidase. Overall, comparison with other plants of the Zingiberaceae family suggested that RZZ possessed moderate antiallergic activity.

### 4.7. Immunomodulatory Activity

Thirty six crude chloroform, methanol and water extracts of 11 medicinal plants commonly used in Thailand were screened for activating human lymphocytes and phagocytes activities [47]. The marker used for detecting lymphocyte activation and proliferation via flow cytometer were CD69 antigen expression while effects on phagocytes were detected by measuring the migration of polymorphonuclear cells towards a chemoattractant under agarose. The chloroform extract of RZZ (CEZZ) significantly stimulated T lymphocytes to express CD69 antigen by 31.6% as compared to the control group (6.2%). The MEZZ and AEZZ gave percentage stimulation of CD69 antigen of 5.8% and 2.3%, respectively. None of the crude extracts stimulated the migration of polymorphonuclear cells but CEZZ and MEZZ did suppress polymorphonuclear cells migration. Phytohemagglutinin (PHA), at the concentration of 5 *μ*g/mL, was used as positive control drug to stimulate the CD69 expression on lymphocytes with an incubation period of 4 h and gave an approximately 46% stimulation of lymphocytes. However, no positive control was used to compare the polymorphonuclear migration rate potential of those extracts.

### 4.8. Antiplatelet Aggregation Activity

The MEZZ, together with another 48 methanol extracts of 37 species of Malaysian medicinal plants, were investigated for their ability to inhibit platelet-activating factor (PAF) receptor-binding effects using rabbit platelets [48]. At the concentrations of 18 *μ*g/mL, the MEZZ produced approximately 96.4% inhibitory effect, which is the highest as compared to the other plants' extracts, with *Boesenbergia pandurata*'s extract being the only plant with closest inhibitory effect (80.4%). Interestingly, the inhibitory effect of MEZZ was greater than Cedrol, a known PAF antagonist (85.2%), which was used as the standard drug. The IC_50_ value obtained for the active MEZZ was 1.2 ± 2.0 *μ*g/mL, which was the lowest compared to Cedrol (2.4 ± 1.3 *μ*g/mL) and *B. pandurate* (8.6 ± 2.6 *μ*g/mL). 

The MEZZ was also reported to exhibit strong antiplatelet aggregation activity at 100 *μ*g/mL in human whole blood *in vitro*, with all extracts exhibiting 100% inhibition [49]. ASA, used as a positive control at the concentration of 25 *μ*g/mL, exhibited inhibitory effects of 100%, 31% and 43% against arachidonic acid-, collagen- and ADP-induced platelet aggregation. Interestingly, compound isolated form RZZ, zerumbone, at the concentration of 100 *μ*g/mL, exerted strong inhibition on platelet aggregation induced by arachidonic acid, collagen and ADP with inhibitory effects of 100%, 68%, and 100%, respectively.

### 4.9. Antioxidant Activity

Fruits and vegetables are known to contain various forms of phytochemicals and antioxidants, particularly polyphenol compounds (e.g., flavonoids and anthocyanins). Their frequent consumption has been associated with a lowered risk of heart disease, cancer, hypertension, and stroke. RZZ, which is a widely used herb taken before meals especially in Fiji, is reported to be the richest source of kaempferol (240 mg/100 g) when compared to another species of Zingiberaceae (*Z. officinale*) [37]. *Z. officinale *contained only trace amount of kaempferol. Other than kaempferol, RZZ contained <1 mg/100 g of myricetin, fisetin and quercetin. Comparison of *Z. officinale*, which possessed total antioxidant capacity (TAC) of 320 mg/100 g and total polyphenol content (TPC) of 200 mg/100 g, also revealed that RZZ contained TAC of 18 mg/100 g and TPC of 130 mg/100 g. For comparison purpose, Trolox C (0.06–0.5 mg/mL), gallic acid (0.1–0.5 mg/mL) and flavonol (5–20 *μ*g/mL), and carotenoids (lycopene and *β*-carotene) (5–20 *μ*g/mL) were used.

### 4.10. Cytotoxic Activity

In an attempt to find plant(s) with potential antitumour promoting agent(s), several medicinal Zingiberaceae rhizomes commonly found in Malaysia were screened using the short-term assay of inhibition of 12-O-tetradecanoyl phorbol-13-acetate- (TPA-) induced Epstein-Barr virus early antigen (EBV-EA) in Raji cells [8]. The assay used to detect the inhibition of TPA-induced EBV-EA was the indirect immunofluorescence assay (IFA) followed by the Western blot technique. For comparison purposed, 1000 *μ*M ascorbic acid, a known antitumour promoter, was used as a standard and exhibited 50% inhibitory effect against TPA-induced EBV-EA activation in Raji cells when compared with negative control. Seven plants' rhizomes (20–640 *μ*g/mL), including RZZ, possessed inhibitory activity towards EBV activation, induced by TPA. The others are of *Curcuma domestica*, *C. xanthorrhiza*, *Kaempferia galanga*, *Z. cassumunar*, *Z. officinale* and *Z. officinale* (red variety). The CEZZ and EEZZ were reported to express EBV (Epstein-Barr virus) activation inhibitory activity when tested at the dose range of 20–160 and 70–320 *μ*g/mL cell culture medium, respectively [8]. Both extracts were also found to cause suppression of 12-O-tetradecanoyl phorbol-13-acetate-(TPA-) induced EBV-EA-D (EBV-early antigen) and EA-R (restricted EA complex) protein production in Raji cells when detected using the Western blot technique. The dose range of CEZZ and EEZZ that exhibited EBV-EA protein suppression was 20–160 and 40–640 *μ*g/mL, respectively. Interestingly, the CEZZ at the above-mentioned range caused complete protein suppression while the EEZZ caused partial protein suppression at 80–160 *μ*g/mL with complete protein suppression seen only at 320 *μ*g/mL. However, the petroleum ether extract of RZZ (PEZZ) was found to be toxic to Raji cells at all concentrations used. Further cytotoxic study on CEZZ and EEZZ using Raji cells indicated that the former was cytotoxic starting from the dose of 320 *μ*g/mL and above (90–100% cells death) while the latter was cytotoxic only at the highest dose used (640 *μ*g/mL) with 60% cells death recorded. The noncytotoxic activity was also seen in the other six plants' rhizomes.

### 4.11. Antiproliferative Activity

Abd Rashid and Lope Pihie [50], upon investigation carried out to determine the effects of extracts and fractions of RZZ on the growth of human breast carcinoma (MCF-7) cell lines, reported that the PEZZ, followed by EAZZ and MEZZ, exhibited antiproliferative activity with EC_50_ value of 4.25 ± 0.05, 8.38 ± 0.08, and 21.31 ± 0.43, respectively. Further partitioning of the MEZZ gave AEZZ and CEZZ with EC_50_ of 85.52 ± 0.70 and 9.52 ± 0.04, respectively, while further extraction of EAZZ yielded EEZZ with EC_50_ value of 59.40 ± 0.42. Based on these findings, a bioassay-guided fractionation was performed on the PEZZ to yield 6 fractions. All fractions were effective against MCF-7 with EC_50_ value of A (11.25 ± 0.21), B (13.60 ± 0.13), C (3.51 ± 0.07), D (11.78 ± 0.17), E (4.07 ± 0.09), and F (10.31 ± 0.12). Fraction C was further fractionated to yield 6 subfractions with only fractions 4 and 5 demonstrating antiproliferative activity against the cancer cells. The EC_50_ value recorded was 2.81 ± 0.24 and 2.49 ± 0.13, respectively. Interestingly, these subfractions were less toxic to normal cells. Comparison was made with the standard chemotherapeutic agent for breast cancer, tamoxifen, which was found to produce EC_50_ value of 4.39 ± 0.21 and 2.56 ± 0.59 against MCF-7 and MDBK cells, respectively.

### 4.12. Antitumour Activity

In another study, Huang et. al. [4] reported on the antitumor activity of diethyl ether extract of RZZ (DEZZ) in cultured P-388D (1) cells by inducing DNA fragmentation in the cells. Study using animal model of P-388D (1)-bearing CDF (1) mice also revealed that the DEZZ could significantly prolong the life of cancer-bearing mice (ILS% = 127.8) at a dosage of 5 mg/kg body weight. 

### 4.13. Antihyperglycemic Activity

Husen et al. [51] carried out the screening of AEZZ, at doses of 50, 100, and 150 mg/kg BW, either subjected to freeze-drying process or not, for potential blood glucose lowering effect in normoglycaemic and streptozotocin-induced hyperglycaemic rats. Comparison with nontreated and 10 mg/kg BW-treated rats revealed that the AEZZ caused no significant reduction in blood glucose level in both groups of rats indicating that the AEZZ did not have antihyperglycemic activity.

### 4.14. Antiamoebic and Antigiardial Activity

The chloroform, methanol, and water extracts of 12 medicinal plants, including *Z. zerumbet*, commonly used by AIDS patients in southern Thailand, were screened for potential anti-amoebic activity [52]. The extracts, at a concentration of 1000 *μ*g/mL, were first tested against *Entamoeba histolytica* strain HTH-56:MUTM and HM1:IMSS growing *in vitro* and extracts that caused inhibition were chosen and retested with concentrations ranging from 31.25 to 1000 *μ*g/mL against *E. histolytica *strain HM1:IMSS. The CEZZ and chloroform extract of *Murraya paniculata*, which exhibited the respective IC_50_ value of 196.9 and 116.5 *μ*g/mL, were classified as being “moderately active.” In comparison, the chloroform extracts of *A. galanga, Barleria lupine, B. pandurata, P. betle and P. chaba*, which produced IC_50_ value ranging from approximately 46 and 91 *μ*g/mL, and methanol extract of *B. pandurata* (IC_50_ of approximately 58 *μ*g/mL), were classified as “active” because their IC_50_ values were less than 100 *μ*g/mL. For validation of methods, metronidazole was used as a standard drug and produced an IC_50_ value of 1.1 *μ*g/mL. 

Various extracts of 12 medicinal plants commonly used by AIDS patients in southern Thailand were screened for potential anti-giardial activity [53]. The chloroform, methanol, and water extracts of the plants, in the doses ranged between 31.25–1000 *μ*g/mL, and a reference drug, metronidazole, in the concentrations ranging between 0.625–20 *μ*g/mL, were tested for their potential to inhibit the *in vitro* growth of *Giardia intestinalis* trophozoites. Despite the chloroform extract of *A. galanga *exerting the highest anti-giardial activity with IC_50_ and MIC values of 37.73 and 125 *μ*g/mL, the CEZZ together with the chloroform extract of *B. pandurata*, *Eclipta prostrata*, *P. betle*, and *P. chaba* and the methanol extract of *B. pandurata* and *E. prostrate* were classified as being active. The CEZZ produced MIC and IC_50_ values of 250 and 69.02 *μ*g/mL, respectively, while the MEZZ and AEZZ showed no activity against *G. intestinalis*. Metronidazole was found to exhibit MIC and IC_50_ value of 2.5 and 0.48 *μ*g/mL, respectively. 

### 4.15. Antimicrobial Activity

Jantan et al. [2] reported that EOZZ exhibited a negative antifungal effect when tested together with another eight species of *Zingiberaceae* using the broth microdilution and the disc gel diffusion methods. The antifungal activity was assessed against five dermatophytes (*Trichophyton mentagrophytes, T. rubrum, Microsporum canis, M. nanum, and Epidermophyton floccosum*), three filamentous fungi (*Aspergillus niger, A. fumigatus and Mucor sp.*) and five strains of yeast (*Saccharomyces cerevisiae, Cryptococcus neoformans, Candida albicans, Ca. tropicalis, and Torulopsis glabrata*). 

Further studies were also carried out on the antibacterial activity of CEZZ, MEZZ, and boiling AEZZ together with another 11 selected Thai medicinal plants used as self-medication by HIV/AIDS patients in Thailand against important pathogenic bacteria commonly associated with AIDS infection, namely, *Staphylococcus aureus*, methicillin-resistant *S. aureus* (MRSA), *Streptococcus mutans*, and *Salmonella typhi *[54] using the paper disc agar diffusion method. The Gram-positive bacteria were proven to be susceptible to CEZZ and chloroform extract of *Alpinia galanga*, *Boesenbergia rotunda*, *Piper betel* and *Spilanthes acmella*, and the methanol extract of *B. rotunda*. Of all tested bacteria, CEZZ, at the concentration of 250 *μ*g/disc, exhibited antibacterial activity against MRSA, *S. aureus* and *Streptococcus mutans* with inhibition zone of 9.5, 9.8, and 8.6 mm, respectively. In comparison to other plants, *A. galanga* was found to exert the highest antibacterial activity followed by *B. rotunda *and* Z. zerumbet*. The MIC and MBC values of CEZZ against those bacteria were not reported in this study. Amikacin (10 *μ*g/disc), ampicillin (10 *μ*g/disc), aztreonam (30 *μ*g/disc), chloramphenicol (30 *μ*g/disc), erythromycin (30 *μ*g/disc), gentamicin (10 *μ*g/disc), kanamycin (30 *μ*g/disc), oxacillin (1 *μ*g/disc), penicillin (10 *μ*g/disc), tetracycline (30 *μ*g/disc), and vancomycin (30 *μ*g/disc) were used as standard reference antibiotics. Only chloramphenicol and vancomycin successfully inhibited the growth of MRSA, *S. aureus* and *S. mutans* with inhibition zone of 24.5, 23.5, and 22.3 mm and 16.7, 16.6, and 21.5 mm, respectively.

In other antibacterial studies using CEZZ, MEZZ, and boiling AEZZ together with 10 selected Thai medicinal plants commonly used to treat infections *against important foodborne pathogenic* bacteria (e.g.,* Bacillus cereus, S. aureus, *MRSA*, Escherichia coli O157:H7, S. Typhi, and Shigella sp.*), only the CEZZ and the chloroform extract of *A. galanga*, *B. rotunda*, *P. betel, *and *Barleria lupulina* exhibited antibacterial activity [55]. CEZZ, which has no effect against Gram-negative bacteria, was reported to be active* against S. aureus* in the paper disc agar diffusion assay. CEZZ was also tested against 17 clinical isolates of *S. aureus*. The MIC and MBC values of CEZZ against most clinical *S. aureus *isolates were 0.79 and >12.5 mg/mL, respectively. CEZZ exerted promising antistaphylococcal activity against clinical isolates PSU037, PSU039, PSU040 and PSU051 with minimum inhibitory concentration (MIC) value of 0.79, 0.79, 0.79, and 0.39 mg/mL, and minimum bactericidal concentration (MBC) value of 12.50, 3.13, 6.25, and 12.50 mg/mL, respectively. CEZZ was also found to significantly inhibit the growth of MRSA and *B. cereus* with similar MIC value of 0.39 mg/mL and MBC values of 3.13 and 1.57 mg/mL. Throughout the study, tetracycline and vancomycin were used as the standard reference drugs. Tetracycline exhibited antibacterial activity against *S. typhi* with MIC and MBC values of approximately 1 and 32 mg/mL, respectively. On the other hand, vancomycin exerted antibacterial activity against *S. aureus *ATCC25923 and MRSA PSU039 with recorded MIC and MBC values of approximately 0.6 and 1.25 mg/mL, and 1.25 and 1.25 mg/mL, respectively. 

### 4.16. Antimycobacterial Activity

In a study carried out to determine the antimycobacterial activity of several medicinal plants, including RZZ, used as self-medication by AIDS patients in Thailand, 38 crude chloroform, methanol and water extracts of 12 plants were tested against *Mycobacterium tuberculosis* using the microplate Alamar blue assay [56]. All plants' extracts tested at the initial concentration of 1000 *μ*g/mL inhibited the growth of *M. tuberculosis* H37Ra. Active extracts were retested at lower concentrations to determine their MICs. Of all RZZ extracts, only the CEZZ and MEZZ were found to produce MICs at 125 and 1000 *μ*g/mL, respectively. However, of all plants tested only the chloroform extract of *A. galanga *and* P. chaba* was considered to have a strong antimycobacterial activity indicated by their MIC value that was <20 *μ*g/mL. As a comparison, rifampin, isoniazid, and kanamycin, which were used as standard drugs, exerted MICs of 0.0023, 0.1, and 2.5 *μ*g/mL, respectively.

### 4.17. Larvicidal Activity

In the first attempt to determine mosquitoes larvicidal activity of RZZ, Tewtrakul et al. [57] investigate the potential of EEZZ and AEZZ, and VOZZ against Anopheline larvae (5–75 *μ*g/mL) and pupae (25–200 *μ*g/mL). Among the three fractions, the EEZZ exerted the most effective larvicidal and pupacidal activities (LD_50_ of 18.9 and 97.4 *μ*g/mL, resp.) while the VOZZ exhibited moderate activities (LD_50_ of 44.1 and 137.7 *μ*g/mL, resp.). Furthermore, at the concentration of 75 *μ*g/mL, the larvicidal effect of EEZZ was approximately 90% compared to the VOZZ, which displayed only 73.3% larvicidal effect. In contrast, at the concentration of 200 *μ*g/mL, both the EEZZ and VOZZ exhibited approximately 70% pupacidal activity while at 25 *μ*g/mL, the EEZZ exhibited 23.3% pupacidal activity as compared to the VOZZ (3.3%). On the other hand, the AEZZ did not show any larvicidal and pupacidal activities (LD_50_ > 2000 *μ*g/mL). The control of this study was 20 *μ*L dimethyl sulfoxide (DMSO) and 30 *μ*L ethanol in 50 mL dH_2_O. However, no attempt was made to compare the effectiveness of RZZ extract and volatile oil with any standard drug.

Recent attempt was made to investigate the chemical compositions and larvicidal potential of essential oils of five edible plants, including *Z. zerumbet*, available in Thailand against mosquito (*Aedes aegypti*) vectors [58]. The analysis of essential oils was carried out using gas chromatography coupled with mass spectrometry (GC/MS) and resulted in the isolation of 20 compounds from EOZZ with *α*-humulene (approximately 31.9%) and zerumbone (approximately 31.7%) being the major components followed by minor quantities of *o*-menth-8-ene (8.46%), santolina triene (5.38%), **β**-caryophyllene (3.36%), and camphor (3.05%). *Citrus hystrix* major components include *d*-limonene, **β**-pinene and terpinen-4-ol; *C. reticulata* major components include* d*-limonene and **γ**-terpinene; Kaempferia galanga major components include ethyl-*p*-methoxycinnamate, pentadecane, and 2-propeonic acid; *Syzygium aromaticum* major components include eugenol and trans-caryophyllene. Assessment of larvicidal efficacy of the essential oils demonstrated that the EOZZ, as well as others, was dose-dependently toxic with different degrees among plant species against both pyrethroid-susceptible and resistant *Ae. aegypti *laboratory strains at LC_50_, LC_95_, and LC_99_ levels. The EOZZ, at the concentrations ranging approximately between 39.2–60.9 ppm, caused percentage of mortality against 4th instar larvae of pyrethroid-susceptible *Ae. aegypti* ranging approximately between 6.3–94.3%. In addition, the recorded LC_50_, LC_95_, and LC_99_ levels for EOZZ were approximately 48.9, 62.2, and 71 ppm. In addition, the LC_50_ value of EOZZ against pyrethroid-resistant strain was 53.1 ppm. As a comparison, *C. reticulate and C. hystrix* were considered to have the most effective larvicidal activity as indicated by their lowest recorded LC_50_, LC_95_, and LC_99_ values (15.4, 36.4, and 58.8 ppm versus 30.1, 51.0, 68.7 ppm, resp.). Furthermore, the susceptibility of the two strains of *Ae. aegypti *to essential oils was slightly different but statistically significant. Despite comparing the essential oils activity with control group, which received only DMSO-distilled water, the authors did not provide comparison with any reference compounds.

## 5. Discussion and Conclusion

*Z. zerumbet* is believed to be native to India and the Malay Peninsula, which corroborate with its rhizomes particular uses as food flavoring and appetizer in Malay and Indian cuisines. Other than that, the RZZ has also been famously used as medicinal herbs in the Asian, Indian, Chinese, and Arabic traditional medicines since ancient times. The claimed medicinal uses of RZZ throughout the world include for the treatment of inflammatory- and pain-associated ailments (i.e., edema, sprain, rheumatism), digestive system-related ailments (i.e., constipation, diarrhea), and skin diseases. Most of these claims have been confirmed via *in vitro* and *in vivo *techniques of biological evaluation.

Traditionally, RZZ was used in various ways. For example, the Malays used either the fresh or boil rhizomes; the Chinese macerated the rhizomes in alcohol; the Indians mixed the rhizome powder with ripe *Morinda citrifolia*, cooked and softened the rhizome or mixed the ground and strained rhizome with water. Based on our literature searches, seven types of extracts, namely, EOZZ/VOZZ, EAZZ, MEZZ, EEZZ, AEZZ, PEZZ, and DEZZ, have been tested for various pharmacological activities using *in vivo* and *in vitro* assays as described above. The AEZZ, EEZZ, and MEZZ indirectly represent the usual way of using the crude extract. For example, the AEZZ exhibited anti-inflammatory and antipyretic activities which could be associated to the Indian traditional medicine uses of mixing the sprained extract to treat stomach ache. On the other hand, the EEOZ has been shown to exert antipyretic and larvicidal activities while MEZZ exhibited antinociceptive, anti-inflammatory activities, which was in line with the Chinese and Taiwanese traditional uses of the rhizome macerated in alcohol and as an anti-inflammatory adjuvant for stomach ache, sprain, and fever. It is worth mentioning that some of the claimed activities in the traditional medicines cured by RZZ and those activities demonstrated by RZZ extracts could be associated with inhibition of PGE synthesis, release or action [6, 7].

The lack of scientific and clinical data in support of better understanding of the efficacy and safety of the herbal drugs has become the major encumbrance to the use of traditional herbal preparations [59]. This is due largely to the negligence of the evaluation of the toxicity and adverse drug reactions of herbal medicines, as they are considered natural and, thus, erroneously, safe. It is therefore pertinent to establish the safety of these preparations through toxicological assessments. In an attempt to study the acute toxicity effect of RZZ, the AEZZ has been reported to be safe at 2000 mg/kg when given orally to broiler chickens [59]. The selection of dosage regime for the *in vivo* studies, which range between 25 and 300 mg/kg, was considered acceptable [60] as they did not exceed the maximum tolerated dose (MTD) suggested for *in vivo *studies. The MTD should not be more than 1000 mg/kg/day [61]. Thus, the AEZZ, EOZZ/VOZZ, MEZZ, and DEZZ can be confirmed to possess *in vivo* antinociceptive, anti-inflammatory, antipyretic, and antitumour activities. It was, however, difficult to link the dosage regime used in all assays to the actual crude drug amount used in traditional medicine as different cultures tend to prepare their medication using different methods of extraction. For example, the Malays used the fresh rhizomes as a cure for stomach ache while the Chinese macerated the rhizomes in alcohol and used it as anti-inflammatory adjuvant to treat stomach ache. On the other hand, the claim of *in vitro* pharmacological activities by various authors need to be analyzed carefully before any conclusion could be made based on the dosage regime used for each of the study. It is generally accepted that any compounds/extracts assayed using *in vitro* techniques should exhibit their activity at EC_50_ or IC_50_ value of less than or equal to 30 *μ*g/mL (≤30 *μ*g/mL) in order to be considered active [62]. Based on the literatures gathered, the selection of dosage regime for *in vitro* studies ranges from 10 to 1000 *μ*g/mL and taking into account that the EC_50_ or IC_50_ must be ≤30 *μ*g/mL, it is plausible to confirm that EAZZ (EC_50_ = 8.4 *μ*g/mL), MEZZ (EC_50_ = 21.3 *μ*g/mL), MEZZ-partitioned CEZZ (EC_50_ = 9.5 *μ*g/mL) and PEZZ (EC_50_ = 4.3 *μ*g/mL) possessed antiproliferative activity; MEZZ possessed antiplatelet aggregation against rabbit platelet (IC_50_ = 1.2 *μ*g/mL); EEZZ possessed larvicidal activity (LD_50_ = 18.9 *μ*g/mL). In contrast, several *in vitro* pharmacological activities were observed at EC_50_ or IC_50_ value that are considered too high and unrealistic (≥30 *μ*g/mL). These include antiplatelet aggregation activity of MEZZ against human platelet (IC_50_ = 100.0 *μ*g/mL) and antimycobacterial (MIC = 1000.0 *μ*g/mL) activities; antiproliferative activity of MEZZ-partitioned AEZZ (EC_50_ = 85.5 *μ*g/mL); antiallergic (IC_50_ = 91.0 *μ*g/mL), antitumour promoter (at dose range of 40–640 *μ*g/mL), antiproliferative (EC_50_ = 59.4 *μ*g/mL), and pupacidal (LD_50_ = 97.4 *μ*g/mL, resp.) activities of EEZZ; antiallergic (IC_50_ = 68.2 *μ*g/mL) and antiproliferative (EC_50_ = 85.5 *μ*g/mL) activities of AEZZ. Other than unrealistic doses producing pharmacological activities as described earlier, some of the activities studied were not properly compared with reference drug. 

In most of the treatment, route of administration of drugs plays an important role and may affect the achievement of the desired pharmacological action of the drugs. Herbal medicines are normally taken via an oral route of administration. However, in some of the preclinical studies to investigate the pharmacological activity of certain medicinal plants, other systemic routes of administration were used (e.g., intraperitoneal, subcutaneous). Based on our literature searches, the antinociceptive and anti-inflammatory activities of RZZ were determined using extracts administered via intraperitoneal or subcutaneous routes. Several reasons for choosing those systemic routes of administration could be suggested. Firstly, the authors tried to compare the effectiveness of antinociceptive activity of extracts with morphine (reference drug), which is clinically given via systemic administration. It is not reasonable to compare the effect of extract with that of reference drug when the extract was given orally (because it was consumed orally) while the reference drug (morphine) was given intraperitoneally. Secondly, since most of the studies were preliminary and aimed at proving the traditional claims of the particular medicinal plant, it is believed that the authors did not establish the pharmacokinetic and pharmacodynamic of the extracts yet. It is known that orally administered compounds/extracts will undergo first-pass hepatic metabolism, and to avoid this from affecting the extract activity, systemic routes of administration were chosen.

In most of the studies, *in vitro* assays were applied to study the extracts pharmacological activities (e.g., antiproliferative, antiplatelet aggregation, and cytotoxicity) because cell lines permit relatively efficient and economical screening for chemotherapeutic activity when compared to *in vivo* assay systems [63]. Although the data obtained using the *in vitro* assays is limited and could not be used as a reliable source to predict what will be obtained in the *in vivo* assays, the former still have the advantage of providing the high-throughput preliminary data in a less time-consuming manner [64]. Other than that, the *in vitro* studies could be used to provide information for narrowing the scope of subsequent investigations which required more comprehensive methods and were time consuming and more expensive [65].

Of all bioactive compound(s) isolated and identified from various types of extracts of RZZ, only zerumbone has been studied extensively. Zerumbone has been demonstrated to possess *in vivo* antinociceptive [66], anti-inflammatory [67] and antitumour [4, 68] activities. While in the *in vitro* studies, zerumbone has been reported to exhibit antiproliferative [69] and antiplatelet aggregation [49] activities. Zederone, isolated from the EEZZ, was suggested to contribute to the larvicidal activity of the extract [42].

In the present paper, we have reviewed the relevant literature to congregate the botanical, ethnobotanical, phytochemical, and pharmacological information on *Z. zerumbet*. Based on the literature survey, *Z. zerumbet *demonstrated various pharmacological activities. However, detail and careful analysis of the reported data leads us to conclude that the plant only possessed promising antinociceptive, anti-inflammatory, antitumour, antiproliferative, antiplatelet aggregation and larvicidal activities. Despite the large number of diseases for which RZZ finds use as a medicine, our critical analysis of the literature revealed that its therapeutic efficacy has been assessed only in a few studies. In view of the wide range of medicinal uses of RZZ in Malay, Chinese, Indian, and Arabic folklores as described in ethnobotanical surveys, it is necessary to conduct more clinical and pharmacological studies at molecular level to investigate untapped potential of this plant. For these reasons, extensive pharmacological and chemical experiments, together with human metabolism, will be a focus for future studies. Recent increase in interest on herbal medicines accompanied by increased laboratory investigation into the pharmacological properties of the bioactive ingredients and their ability to treat various diseases has contributed to numerous drugs/herbal extracts entering the international market. As the recent information shows, it is also possible that zerumbone might be useful in the development of new drugs to treat pain- and inflammation-associated ailments and cancer. However, clinicians should remain alert until more definitive studies demonstrate the safety, efficacy and quality of the compound. Last but not the least, this paper emphasizes the potential of *Z. zerumbet *to be employed in the development of new therapeutic drugs and provide the basis for future research on the application of transitional medicinal plants.

## Abbreviations

RZZ: Rhizome of *Z. zerumbet *
EOZZ: Essential oil of RZZ
MEZZ: Methanol extract of *Z. zerumbet *
ALT: Alanine transaminase
ALP: Alkaline phosphatase
AST: Aspartate transaminase
EEZZ: Ethanol extract of RZZ
AEZZ: Aqueous extract of RZZ
VOZZ: Volatile oil of RZZ
PEZZ: Petroleum ether extract of RZZ
DEZZ: Diethyl ether extract of RZZ
MRSA: Methicillin-resistant *S. aureus* (MRSA)
MIC: Minimum inhibitory concentration
MBC: Minimum bactericidal concentration
DMSO: Dimethyl sulfoxide
MTD: Maximum tolerated dose.
