# Corrigendum to “*Zingiber zerumbet* (L.) Smith: A Review of Its Ethnomedicinal, Chemical, and Pharmacological Uses”

**DOI:** 10.1155/2016/8621824

**Published:** 2016-07-04

**Authors:** N. J. Yob, S. Mohd. Jofrry, M. M. R. Meor. Mohd. Affandi, L. K. Teh, M. Z. Salleh, Z. A. Zakaria

**Affiliations:** ^1^Department of Pharmaceutics, Faculty of Pharmacy, MARA University of Technology, Puncak Alam Campus, Bandar Puncak Alam, 42300 Selangor, Malaysia; ^2^Pharmacogenomics Center, Faculty of Pharmacy, MARA University of Technology, Shah Alam, 40450 Selangor, Malaysia; ^3^Department of Biomedical Sciences, Faculty of Medicine and Health Science, Putra Malaysia University, UPM Serdang, 43400 Selangor, Malaysia

The article titled “*Zingiber zerumbet* (L.) Smith: A Review of Its Ethnomedicinal, Chemical, and Pharmacological Uses” [1] has been corrected as the caption of one of the figures was incorrect. The original caption read “Figure 1: The leaves and inflorescences of* Z. zerumbet*.” The correct caption text should have read “Figure 1: The leaves and inflorescences of* Z. zerumbet*. (Figure reproduced with the permission of http://www.bambooland.com.au/gingers/zingiber-zerumbet).” The new caption has been corrected in place.

## References

[1] Yob N. J., Mohd Jofrry S., Meor Mohd Affandi M. M. R., Teh L. K., Salleh M. Z., Zakaria Z. A. (2011). *Zingiber zerumbet* (L.) Smith: a review of its ethnomedicinal, chemical, and pharmacological uses. *Evidence-Based Complementary and Alternative Medicine*.

